# Does tranexamic acid have a positive effect on the outcome of older multiple trauma patients on antithrombotic drugs? An analysis using the TraumaRegister DGU^®^

**DOI:** 10.3389/fmed.2024.1324073

**Published:** 2024-02-20

**Authors:** Stefanie Fitschen-Oestern, Georg Maximilian Franke, Nora Kirsten, Rolf Lefering, Sebastian Lippross, Ove Schröder, Tim Klüter, Michael Müller, Andreas Seekamp, TraumaRegister DGU

**Affiliations:** ^1^Department of Trauma Surgery, University Medical Center of Schleswig-Holstein, Kiel, Germany; ^2^Department of Trauma Surgery, Hannover Medical School, Hannover, Germany; ^3^Institute for Research in Operative Medicine (IFOM), University Witten/Herdecke, Cologne, Germany

**Keywords:** multiple trauma, TraumaRegister DGU^®^, hemorrhage, anticoagulation as premedication, tranexamic acid

## Abstract

**Background:**

Acute hemorrhage is one of the most common causes of death in multiple trauma patients. Due to physiological changes, pre-existing conditions, and medication, older trauma patients are more prone to poor prognosis. Tranexamic acid (TXA) has been shown to be beneficial in multiple trauma patients with acute hemorrhage in general. The relation of tranexamic acid administration on survival in elderly trauma patients with pre-existing anticoagulation is the objective of this study. Therefore, we used the database of the TraumaRegister DGU^®^ (TR-DGU), which documents data on severely injured trauma patients.

**Methods:**

In this retrospective analysis, we evaluated the TR-DGU data from 16,713 primary admitted patients with multiple trauma and age > =50 years from 2015 to 2019. Patients with pre-existing anticoagulation and TXA administration (996 patients, 6%), pre-existing anticoagulation without TXA administration (4,807 patients, 28.8%), without anticoagulation as premedication but TXA administration (1,957 patients, 11.7%), and without anticoagulation and TXA administration (8,953 patients, 53.6%) were identified. A regression analysis was performed to investigate the influence of pre-existing antithrombotic drugs and TXA on mortality. A propensity score was created in patients with pre-existing anticoagulation, and matching was performed for better comparability of patients with and without TXA administration.

**Results:**

Retrospective trauma patients who underwent tranexamic acid administration were older and had a higher ISS than patients without tranexamic acid donation. Predicted mortality (according to the RISC II Score) and observed mortality were higher in the group with tranexamic acid administration. The regression analysis showed that TXA administration was associated with lower mortality rates within the first 24 h in older patients with anticoagulation as premedication. The propensity score analysis referred to higher fluid requirement, higher requirement of blood transfusion, and longer hospital stay in the group with tranexamic acid administration. There was no increase in complications. Despite higher transfusion volumes, the tranexamic acid group had a comparable all-cause mortality rate.

**Conclusion:**

TXA administration in older trauma patients is associated with a reduced 24-h mortality rate after trauma, without increased risk of thromboembolic events. There is no relationship between tranexamic acid and overall mortality in patients with anticoagulation as premedication. Considering pre-existing anticoagulation, tranexamic acid may be recommended in elderly trauma patients with acute bleeding.

## Introduction

Uncontrolled hemorrhage is one of the leading causes of mortality and morbidity in multiple trauma patients worldwide (1). A high number of trauma patients with bleeding present a coagulopathy on hospital admission (2). The presence of coagulopathy is associated with an increased incidence of multiple organ failure (3).

According to the national S3 guideline, the administration of tranexamic acid (TXA) in multiple trauma patients with massive bleeding is recommended. Several studies have shown that tranexamic acid administration reduces the risk of mass transfusion and mortality in trauma patients (4–6). Especially in patients with acute bleeding, the risk of death can be safely reduced (6). TXA blocks the formation of plasmin by inhibiting the proteolytic activity of plasminogen activators. This inhibits plasmin in its ability to lyse fibrin (7).

However, TXA is rarely used due to the risk of thrombosis in some patient groups (8, 9). Especially if not all pre-existing conditions and medications are known, as in a preclinical emergency setting, there are still reservations about the administration of TXA. Most studies examine polytrauma patients in general but do not focus separately on high-risk groups.

Along with the aging population, multiple trauma in the elderly has increased over the last few decades (10). Reduced physiological reserve and the existence of multiple medical comorbidities present additional challenges to management (10). In contrast to younger trauma patients, elderly patients experience significantly higher mortality rates and complications after multiple traumas (11).

The following study aims to evaluate the administration of TXA in the emergency room management of older multiple trauma patients with pre-existing anticoagulation. We used the TraumaRegister DGU^®^ to evaluate if the administration of TXA is associated with higher survival rates in elderly trauma patients with anticoagulation as premedication and if there is a higher frequency of complications such as thromboembolic events after TXA administration.

## Methods

### TraumaRegister DGU^®^

The TraumaRegister DGU^®^ (TR-DGU) of the German Trauma Society (Deutsche Gesellschaft für Unfallchirurgie, DGU) was founded in 1993. The aim of this multi-center database is the pseudonymized and standardized documentation of severely injured patients.

Data are collected prospectively in four consecutive time phases from the site of the accident until discharge from the hospital: (A) prehospital phase, (B) emergency room and initial surgery, (C) intensive care unit, and (D) discharge. The documentation includes detailed information on demographics, injury patterns, comorbidities, pre- and in-hospital management, a course on intensive care unit, and relevant laboratory findings including data on transfusion and outcome of each individual. The inclusion criterion is admission to the hospital via the emergency room with subsequent ICU/ICM care or reaching the hospital with vital signs and dying before admission to the ICU.

The infrastructure for documentation, data management, and data analysis is provided by the AUC—Academy for Trauma Surgery (AUC—Akademie der Unfallchirurgie GmbH)—a company affiliated to the German Trauma Society. Scientific leadership is provided by the Committee on Emergency Medicine, Intensive Care and Trauma Management (Sektion NIS) of the German Trauma Society. Participating hospitals submit their data pseudonymized into a central database via a web-based application. Scientific data analysis is approved according to a peer review procedure laid down in the publication guideline of the TraumaRegister DGU^®^.

The participating hospitals are primarily located in Germany (90%), but a growing number of hospitals from other countries contribute data as well (at the moment from Austria, Belgium, China, Finland, Luxembourg, Slovenia, Switzerland, Netherlands, and the United Arab Emirates). Currently, over 28,000 cases from almost 700 hospitals are entered into the database per year. Participation in the TraumaRegister DGU^®^ is voluntary. For hospitals associated with the TraumaNetzwerk DGU^®^, however, the entry of at least a basic data set is obligatory for reasons of quality assurance.

### Study cohort

**Figure 1:**
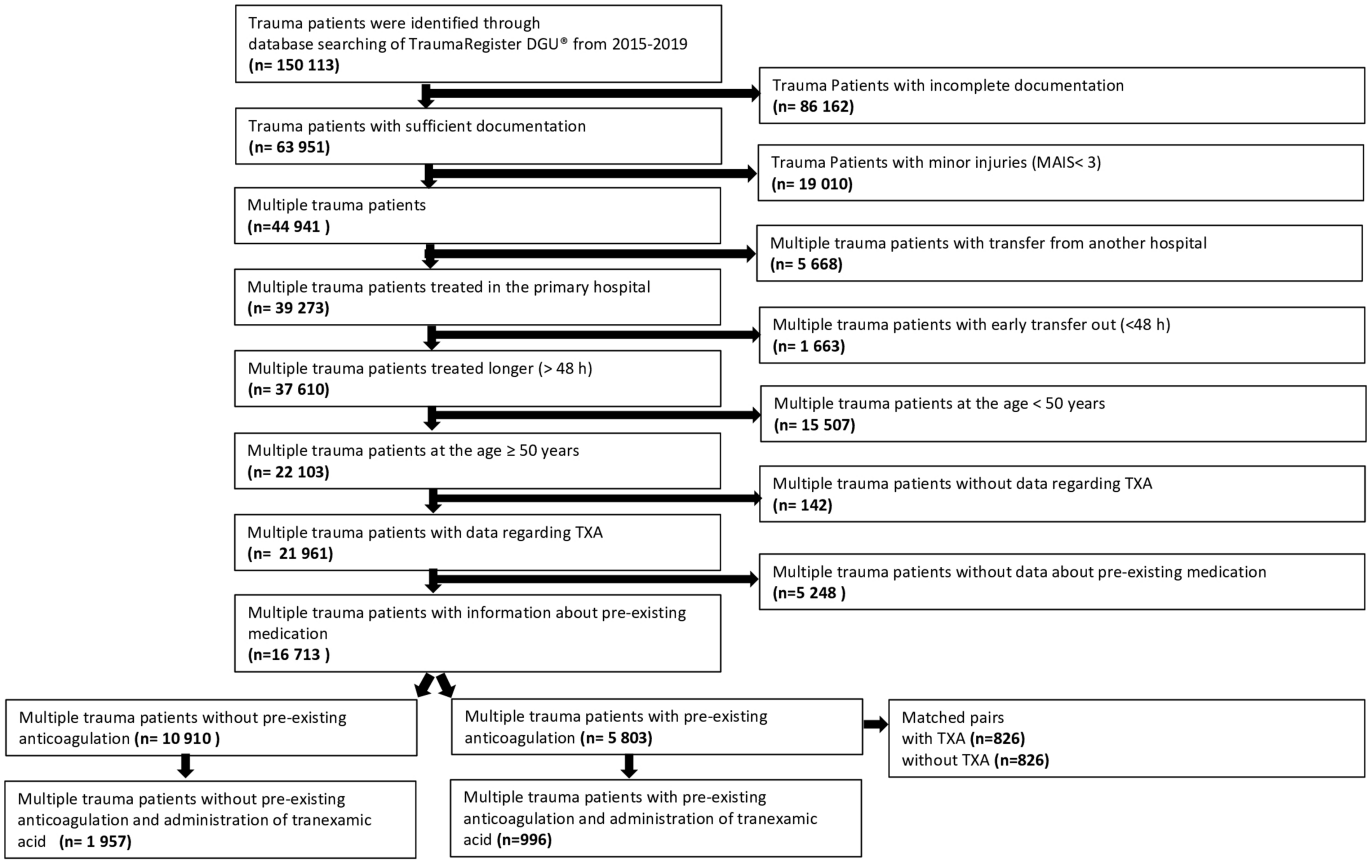
Patient selection flow chart of multiple trauma patients with pre-existing anticoagulation and tranexamic acid administration. Multiple trauma patients at the age >50 years and pre-existing anticoagulation were analyzed regarding tranexamic acid administration during trauma care. For the propensity score study, multiple trauma patients with anticoagulation as premedication and tranexamic acid administration were matched with multiple trauma patients with pre-existing anticoagulation without tranexamic acid administration.

Primary admitted patients who were treated in Germany between 2015 and 2019 were included (Figure 1). Further including criteria were age ≥ 50 years, and the worst injury severity level according to the Abbreviated Injury Scale should be 3 or more (MAIS 3+).

Patients treated at local (level 3), regional (level 2), and supra-regional (level 1) trauma centers and whose treatment was documented with the complete dataset were included in the evaluation. The centers are classified in the TraumaNetzwerk DGU^®^ according to the level of care (level I, II, and III) within the German healthcare system (12).

Patients documented with the basic dataset only were excluded since TXA administration was missing. Patients with incomplete data regarding pre-existing anticoagulation were excluded as well. Only primary admissions were considered without transfer-in patients (no data about prehospital TXA) and early transfers out (no outcome data). A total of 16,713 patients qualified for this investigation. Patients were divided into two groups depending on the intake of antithrombotic drugs prior to admission. Again, two subgroups were formed based on the administration of tranexamic acid.

### Variables

TXA administration has been documented both in the prehospital setting as well as in the emergency room within 3 h after trauma. Tranexamic acid 0.5–1 g was administered slowly intravenously as an injection solution. It was documented whether tranexamic acid was given preclinically, at the emergency room, or preclinically and at the emergency room. The exact time of administration within 3 h after trauma was not documented.

The outcome was defined as in-hospital mortality, mortality within 24 h after admission, the requirement of blood transfusion until intensive care unit (ICU) admission, hospital stay, stay at ICU, and occurrence of thromboembolic events.

Pre-existing anticoagulation prior to admission was defined as the regular intake of either antiplatelet drugs, vitamin K antagonists, direct oral anticoagulation, or heparinoids. The pre-existing anticoagulation was taken regularly. The information on anticoagulation as prior medication was provided by the patients, relatives, and the general practitioner. Single doses were not included.

The Revised Injury Severity Score II (RISC II) was applied as a prognostic parameter. The RISC II score is validated for risk of death prediction in severely injured patients. Calculation includes type and severity of injury, mechanism of trauma, age, sex, ASA score, pupil reaction and size, motor function, blood pressure, and laboratory parameters such as INR, base excess, hemoglobin, and cardio-pulmonary resuscitation (13).

### Study approval

The presented study was approved by the local ethics committee of the medical faculty of Kiel University (D491/21). The publication is in line with the publication guidelines of the TraumaRegister DGU^®^ and registered as TR-DGU project ID-2020-043.

### Statistical methods

Statistical analyses were performed with SPSS 24.0 (IBM, Armonk, NY, United States). Continuous and categorical variables are presented as mean with standard deviation (SD) or as numbers (percentages), respectively. Expected mortality was calculated based on the Revised Injury Severity Classification score, version II (RISC II). This score combines 13 different early prognostic factors available shortly after admission. It has been developed and validated with TR-DGU data (13). Multivariable analyses using logistic regression models were performed to identify the adjusted effects of TXA administration on hospital mortality. In addition to the RISC II score, further adjustments were made for the trauma center level of care and pre-existing anticoagulation. Results are presented as odds ratios (OR) with 95% confidence intervals (95% CI). To assess the independent impact of tranexamic acid in patients with anticoagulation as premedication, a propensity score matching was performed. A logistic regression model was used to determine the propensity score, which is the probability of TXA administration (Table 1). Patients with and without TXA administration were then matched according to the propensity score (± 1%). In total, 5,482 patients were available for propensity score matching. Patients with and without tranexamic acid administration were matched. A total of 826 pairs were found. Outcome data were then compared using Pearson’s chi-squared test. A significance level of *p* < 0.05 was applied.

**Table 1:** Mean age, injury severity score, RISC II score and observed mortality of multiple trauma patients with and without anticoagulant therapy (*n* = 16,713).

	No anticoagulation as premedication (NA)			Anticoagulation as premedication (A)		
	No TXA 8,953 (53.6%)	TXA 1,957 (11.7%)	Total 10,910 (65.3%)	No TXA 4,807 (28.8%)	TXA 996 (6%)	Total 5,803 (34.7%)
Age, years, mean (SD)	65.3 (11.6)	63.0 (10.5)	64.9 (11.4)	77.1 (10.0)	75.1 (10.5)	76.8 (10.1)
ISS, points, mean (SD)	19.5 (9.6)	28.1 (13.5)	21.0 (10.9)	20.0 (9.3)	25.9 (12.2)	21.0 (10.1)
Expected mortality based on RISC II (%)	9.0	20.1	11.0	23.6	34.7	25.5
Hospital mortality, (%), mean	9.8	20.2	11.7	26.1	36.8	28.0
Blood transfusion (%), mean	3.2	33.4	8.6	3.4	32.5	8.4

ISS: Injury severity score, RISC II revised injury severity classification version 2.

## Results

A total of 16,713 patients at the age of ≥50 years could be included in this study. In total, 2,953 patients (17.7%) received tranexamic acid (Figure 1). A distinction was made between four groups: patients with pre-existing anticoagulation and administration of tranexamic acid (996 patients, 6%), patients without pre-existing anticoagulation and administration of tranexamic acid (1,957 patients, 11.7%), patients with pre-existing anticoagulation without tranexamic acid (4,807 patients, 28.8%), and patients without both pre-existing anticoagulation and administration of tranexamic acid (8,953 patients, 53.6%).

In summary, 13,760 patients did not receive tranexamic acid (82.3%), 948 patients received tranexamic acid preclinically (5.7%), 1,700 patients received tranexamic acid at the emergency room (10.2%), and 305 patients received tranexamic acid preclinically and at the emergency room (1.8%). Concerning 2,953 patients who received tranexamic acid, 32.1% received tranexamic acid preclinically, 57.6 patients received tranexamic acid at the emergency room, and 10.3% received tranexamic acid preclinically and/or at the emergency room.

Pre-existing coagulation disorders based on regular intake of antithrombotic drugs were present in 5,803 patients (35%, Figure 1). The antithrombotic drugs used by these patients were acetylsalicylic acid (53.7%), direct oral anticoagulants (20.8%), vitamin K antagonists (21.9%), or heparin (2.7%). Trauma patients with pre-existing anticoagulation received tranexamic acid in 996 cases (17.2%) and patients without pre-existing coagulation disorders received tranexamic acid in 1,957 cases (17.9%).

First, we compared patients with and without pre-existing anticoagulation (Table 2). Patients without anticoagulation as premedication (NA) were younger than patients with pre-existing anticoagulation (A) (average age 65 versus 77 years). Patients who received tranexamic acid were younger in both patient collectives (Table 2).

**Table 2:** Multivariable analysis using a logistic regression model with overall death as a dependent variable of trauma patients with or without anticoagulation before admission (*n* = 16,713).

	Regression coefficient	Standard error	Odds ratio (95% CI)	*p*-value
RISC II score	−0.874	0.016	0.42 (0.40–0.43)	<0.001
Hospital level of care				
Level 1	−0.06	0.07	0.94 (0.82–1.09)	0.411
Level 2	−0.27	0.18	0.76 (0.54–1.08)	0.129
Anticoagulative therapy	0.183	0.056	1.20 (1.08–1.34)	0.001
Administration of TXA	−0.042	0.067	0.96 (0.84–1.09)	0.528

The influence of level of trauma center, pre-existing anticoagulation, and administration of tranexamic acid on death was evaluated. RISC II: revised injury severity classification version 2, TXA: tranexamic acid.

The average ISS was 21.0 in both patient groups, but patients with tranexamic acid administration had a significantly higher ISS (NA: 28.1, A: 25.9). RISC II was significantly higher in the patient group with anticoagulation as premedication (23.6) than in patients without anticoagulation (9). Patients who received tranexamic acid achieved generally a higher RISC II (NA: 20.1, A: 34.7). Hospital mortality was higher in the group with anticoagulation as premedication, both with (36.8) and without tranexamic acid (26.1). The proportion of patients who received blood transfusions was 10 times higher in both patient groups with tranexamic acid administration (Table 2).

To investigate the relationships between pre-existing anticoagulation and tranexamic acid administration on all-cause mortality, we performed a regression analysis with trauma patients with and without anticoagulation at the age of ≥50 years. The administration of tranexamic acid was not associated with lower mortality in the whole patient collective (OR 0.96, 95% CI 0.84–1.09) (Table 3), but the administration of tranexamic acid had a positive effect on mortality within 24 h: OR = 0.84 (0.71–0.99) (*p* = 0.041) (Table 4). The presence of pre-existing anticoagulation is more likely to cause death within 24 h (OR = 1.28 (1.10–1.48) *p* = 0.001).

**Table 3:** Multivariable analysis using a logistic regression model with death within 24 hours after admission as a dependent variable of trauma patients with or without anticoagulation before admission (*n* = 16,713).

	Regression coefficient	Standard error	OR (95% CI)	*p*-value
RISC II score	−0.79	0.02	0.45 (0.44–0.47)	<0.001
Hospital level of care				
Level 1	−0.18	0.11	0.84 (0.68–1.03)	0.093
Level 2	0.20	0.24	1.22 (0.76–1.96)	0.405
Anticoagulative therapy	0.24	0.08	1.28 (1.10–1.48)	0.001
Administration of TXA	−0.18	0.09	0.84 (0.71–0.99)	0.041

The influence of level of care, pre-existing anticoagulation, and tranexamic acid administration on death within 24 hours after trauma was examined. RISC II: revised injury severity classification version 2, TXA: tranexamic acid.

**Table 4:** Multivariable analysis using a logistic regression model with death during hospital stay (A) death within 24 hours (B) as a dependent variable only of trauma patients with anticoagulation as premedication (*n* = 5,803).

	Regression coefficient	Standard error	OR (95% CI)	*p*-value
(A)				
RISC II score	−0.84	0.02	0.43 (0.41–0.45)	<0.001
Hospital level of care				
Level 1	−0.08	0.1	0.92 (0.76–1.11)	0.39
Level 2	−0.26	0.23	0.77 (0.49–1.22)	0.27
Administration of TXA	−0.01	0.1	0.99 (0.83–1.2)	0.95
(B)				
RISC II score	−0.81	0.03	0.44 (0.2–0.47)	<0.001
Hospital level of care				
Level 1	−0.13	0.14	0.88 (0.67–1.14)	0.32
Level 2	0.34	0.30	1.41 (0.78–2.56)	0.26
Administration of TXA	−0.26	0.12	0.77 (0.61–0.98)	0.03

The influence of level of care and tranexamic acid administration on death was examined.

A regression analysis was performed only for multiple trauma patients (at the age of ≥50 years) with pre-existing anticoagulation. Neither trauma center level of care nor tranexamic acid administration showed an effect on all-cause mortality (tranexamic acid OR = 0.99 (0.83–1.2) (*p* = 0.95) (Table 1, A). Administration of tranexamic acid showed a positive effect on 24 h-mortality of patients with pre-existing anticoagulation OR = 0.77 (0.61–0.98) (*p* = 0.05) (Table 1, B).

In order to better compare patients with pre-existing anticoagulation, propensity score matching was performed in patients with and without tranexamic acid administration (*n* = 5,482). Matching was performed considering different variables listed in Table 5. A total of 826 pairs of patients could be found with identical propensity scores (= probability to receive TXA).

**Table 5:** Multivariate logistic regression analysis with‚ prehospital TXA’ as dependent variable, in patients with anticoagulation therapy before admission (*n* = 1,652).

	Regression coefficient	Standard error	Odds ratio
Age ≥ 60 years	−0.155	0.137	0.86
AIS abdomen ≥3	0.421	0.150	1.52
AIS extremities ≥3	0.336	0.101	1.40
ISS, per point	0.023	0.004	1.02
Isolated trauma	−0.142	0.107	0.87
Penetrating injury	0.712	0.224	2.04
Systolic blood pressure < 100 mmHg	0.360	0.107	1.43
Helicopter transport	0.497	0.091	1.64
Prehospital treatment			
Intubation	0.171	0.100	1.19
Chest tube	−0.810	0.243	2.36
Pelvic binder	0.775	0.147	2.17
CPR	−0.810	0.229	0.45
I.v. fluid administration (reference: unknown)			
Up to 500 mL	0.059	0.165	1.06
Up to 1,000 mL	0.486	0.173	1.60
Up to 2,000 mL	0.815	0.197	2.26
> 2,000 mL	1.252	0.411	3.50
I.v. fluids 2	0.486	0.173	1.60
I.v. fluids 3	0.815	0.197	2.26
I.v. fluids 4	1.252	0.411	3.50
Hemoglobin ≤ 8 g/dl	0.749	0.162	2.11
Catecholamines	0.929	0.097	2.53
Level 1 trauma center	−0.237	0.114	0.79
Level 2 trauma center	−1.139	0.376	0.32

The calculated probabilities for receiving TXA (rounded percentages) were used as propensity score for the following matching. TXA: tranexamic acid, ASA: American society of anesthesiologists, ISS: injury severity score, SBP: systolic blood pressure, INR: international normalized ratio, i.v.: intravenous, ER: emergency room, Prbc: packed red blood cells, FFP: fresh frozen plasma, RISC II: revised injury severity classification version 2, ICU: intensive care unit, LOS: length of stay.

Table 6 presents the data for those matched pairs of propensity scores. Slightly more men received TXA (TXA: 566 (68.5%), No TXA: 519 (62.8%), *p* = 0.015). In the emergency room, obvious differences occurred for volume administration (TXA: 1,532 ± 1727, No TXA: 959 ± 1,097, *p* < 0.001) and blood transfusion (TXA: 218 (26.4%), No TXA: 74 (9%), <0.001).

**Table 6:** We performed propensity score matching in patients with anticoagulation as premedication.

	TXA administered	No TXA	*p*-value
	*N* = 826	*N* = 826	
Age, years, mean (SD)	75.6 (10.2)	76.1 (10.1)	0.367
Male sex, *n* (%)	566 (68.5)	519 (62.8)	0.015
ASA classification 3/4, *n* (%)	475 (59.6)	486 (61.9)	0.346
Anticoagulant before admission:			
Antiplatelet agents, *n* (%)	427 (51.7)	446 (54.0)	0.349
Vitamin K antagonists, *n* (%)	200 (24.2)	170 (20.6)	0.077
Direct oral anticoagulants, *n* (%)	168 (20.3)	178 (21.5)	0.545
Parenteral anticoagulants, *n* (%)	21 (2.5)	22 (2.7)	0.877
Blunt trauma, *n* (%)	774 (96.6)	778 (96.2)	0.994
ISS, points, mean (SD)	24.4 ± 11.1	23.8 ± 12.0	0.309
SBP preclinical, mmHg, mean ± SD	137.4 ± 39.1	136.3 ± 37.8	0.582
SBP at ER, mmHg, mean ± SD	130.4 ± 37.4	129.2 ± 35.2	0.505
Hemoglobin, g/dL, mean ± SD	11.8 ± 2.5	12.0 ± 2.4	0.044
Base excess, mmol/L, mean ± SD	−2.1 ± 5.1	−2.0 ± 5.2	0.679
INR, mean ± SD	1.6 ± 1.0	1.5 ± 0.8	0.029
I.v. fluids prehospital, mL, mean ± SD	762 ± 511	766 ± 514	0.879
I.v. fluids at ER, mL, mean ± SD	1,532 ± 1,727	959 ± 1,097	<0.001
Expected mortality based on RISC II (mean)	32.4%	31.0%	0.404
ICU LOS, days, median (IQR)	7 (2–16)	4 (1–12)	<0.001
Hospital LOS, days, median (IQR)	14 (6–25)	12 (4–22)	0.006
24 h mortality, *n* (%)	120 (14.5)	140 (16.9)	0.177
Hospital mortality, *n* (%)	294 (35.6)	274 (33.2)	0.300
Blood transfusion before ICU admission, *n* (%)	218 (26.4)	74 (9.0)	<0.001
Mass transfusion (≥ 10 pRBC) (%)	18 (2.2)	2 (0.2)	<0.001
Thromboembolic event, *n* (%)	26 (3.3)	24 (3.1)	0.823
Unconsciousness (GCS 3–8)	253 (31.5%)	240 (30.7%)	0.745
Prehospital CRP (cardiac arrest)	28 (3.4%)	29 (3.5%)	1.00

Patients with tranexamic acid administration were matched with patients without tranexamic acid administration. We investigated the effect of tranexamic acid administration in patients with comparable injury severity. Study outcome after propensity matching (*n* = 1,652) is shown in Table 5. TXA tranexamic acid, pRBC packed red blood cells.

There were minor differences with regard to RISC II (TXA: 32.4 ± 31.3, No TXA 31 ± 32.9, *p* = 0.404), mortality within 24 h [TXA: 120 (14.5%), No TXA: 140 (16.9), *p* = 0.177], and all-cause mortality [TXA: 294 (35.6%), No TXA: 274 (33.2%), *p* = 0.300]. Differences were not significant.

Hospital stay [TXA: 14 (6-24), No TXA: 12 (4-22), *p* = 0.006] and ICU length of stay [TXA:7 (2-16), No TXA: 4 (1-12), *p* < 0.001] were significantly longer for patients who received tranexamic acid (Table 6). We did not find a relevant difference in thromboembolic complications [TXA:26 (3.3%), No TXA 24 (3.1%), *p* = 0.823].

## Discussion

Acute uncontrolled bleeding remains one of the most common causes of death after severe injuries (14). Tolerance to extended blood loss in older patients is limited due to reduced physiological reserve (15, 16).

Blood loss causes hypoperfusion, which leads to tissue damage, immune response, and activation of the coagulation system, resulting in trauma-associated coagulopathy (17). Bleeding-associated coagulopathy correlates, in turn, with the development of organ failure (17).

A key component of trauma-induced coagulopathy represents systemic fibrinolysis (18). Tranexamic acid, an inhibitor of the fibrinolysis system, can reduce blood loss in trauma patients (6). Several studies have documented that tranexamic acid administration reduces mortality in trauma patients without increasing the risk of thromboembolic complications (19, 20). There is little data to date on the effect of tranexamic acid in patients with pre-existing conditions and prior medication.

Depending on the study and patient population, tranexamic acid was used in 10–15% of included multiple trauma patients (4). In the study of Curry et al., only 6% of trauma patients with coagulation disorders and 11.7% without coagulation disorders received tranexamic acid as medication, which seems low considering acute hemorrhage is responsible for 40% of mortality in polytrauma patients (21).

According to manufacturer’s guidelines, tranexamic acid should not be administered to patients with certain coagulation disorders, consumptive coagulopathy, renal disease, and known seizures. Several preconditions in combination with tranexamic acid administration are associated with a high risk of complications (22, 23). Limited data on trauma patients with special pre-existing conditions and premedication might cause restrained use of tranexamic acid. Increased age of the population has led to a rise in bleeding trauma patients with pre-existing anticoagulation (24). Depending on age and comorbidities, several changes in coagulation such as fibrinogen rise, factor VIII, and VWF rise are found, some fibrinolysis markers increase, and platelets are more active (25, 26). Such patients are not treated uniformly, even in major trauma centers.

In our evaluation, polytrauma patients, both with and without anticoagulation as premedication, who received tranexamic acid were younger and more severely injured than patients without tranexamic acid. Blood transfusion, RISC II, and mortality rate were significantly higher in the groups with tranexamic acid due to patients’ age and overall conditions (Table 2). Imach et al. evaluated trauma patients from the TraumaRegister DGU^®^ with and without administration of tranexamic acid without age restriction and showed comparable results in terms of age and injury severity (4). In most evaluations, tranexamic acid was used more in younger patients with hemodynamic instability than in older patients (27, 28). RISCII and the mortality rate of the tranexamic acid group without anticoagulation were comparable with other evaluations (4). RISC II and the mortality rate of patients with anticoagulation as premedication and tranexamic acid were significantly higher than the group without anticoagulation as premedication (Table 2). RISC II includes worst and second-worst injury, age, INR, blood pressure, hemoglobin, and ASA (13). Higher ASA scores, low hemoglobin, and low blood pressure cause higher RISCII and mortality in patients with pre-existing anticoagulation.

Pre-existing coagulation disorders made mortality likely after trauma, while administration and timing of tranexamic acid within the first 3 h had no significant effect on total mortality when all patients at the age of ≥50 years were included. Considering the mortality within the first 24 h after trauma, death became more likely with pre-existing anticoagulation while the administration of tranexamic acid made death less likely.

Our results correlated with previous findings of patients without age limitation, which demonstrated a reduction in mortality with the administration of tranexamic acid in the first hours after trauma (4, 29). A relation in all-cause mortality in patients receiving anticoagulation as premedication could not be demonstrated in our regression analysis after tranexamic acid administration. Regarding the associations of tranexamic acid with total mortality, there seems to be a great variability depending on the study population, medical care, and pre-existing conditions (30). Using sensitivity analysis, Karl et al. demonstrated reduced 1-month mortality after tranexamic acid administration in the context of a meta-analysis (30). Depending on injury severity and timing of tranexamic acid administration, Neeki et al. demonstrated a reduction in all-cause mortality (31). The combination of injuries may also play a role in the associations of tranexamic acid with all-cause mortality. A reduction in all-cause mortality could not be detected in patients with severe brain injuries.

Result heterogeneity is caused by patient characteristics, such as injury severity, since not all evaluations examined patient groups of comparable age, similar injuries, and injury severity (30).

The timing and dosage of tranexamic acid also play a role. Tranexamic acid administration has an early antifibrinolytic effect within 4 h after trauma (32). Administration of tranexamic acid treatment within 3 h of injury reduces the risk of hemorrhage death by approximately one-third (19, 23). The benefit of tranexamic acid administration decreased by 10% for every 15 min of treatment delay until 3 h after injury, when there was no benefit (33).

Additionally, due to the manufacturer guidelines, the elimination half-life of tranexamic acid is approximately 3 h. After intravenous administration of 10 mg/kg body weight, approximately 90% of tranexamic acid is excreted within the first 24 h. Therefore, the effect of tranexamic acid is limited by time (34).

Older trauma patients are known to have higher rates of complications after multiple trauma than young patients (35). Pre-existing medical conditions have an impact on mortality rate but lose their effect with increasing injury severity (36). For better comparability, a propensity score matching was performed to compare patients with pre-existing anticoagulation at the same age with similar injury severity.

Lower mean hemoglobin concentration and higher mean INR (international normalized ratio) were demonstrated in the group with tranexamic acid administration. The INR and Hb (hemoglobin) are variables that are included in RISC II (13). We could not find a significant difference in RISC II between both groups.

Patients of the tranexamic acid group demonstrated higher blood loss, which probably led to the administration of tranexamic acid. As a result of higher blood loss, blood transfusions were given more frequently in the group with tranexamic acid administration (Table 6). Blood transfusion and mass transfusion are often associated with more medical interventions, longer hospital stay, and higher mortality (37). Patients with tranexamic acid administration had a longer hospital stay and a longer stay at ICU. Transfusion of blood products and the number of transfused units show a correlation with thromboembolic events (38). Although transfusion of blood and mass transfusion were significantly higher in the group receiving tranexamic acid, no more thromboembolic events occurred than in the group without tranexamic acid administration. Therefore, older trauma patients with anticoagulation as premedication do not show more complications after tranexamic acid administration, just like younger multiple trauma patients with tranexamic acid administration (39).

Bleeding and mass transfusions are associated with an increase in mortality (37). Significant higher mass transfusion in the tranexamic acid group did not cause higher mortality than patients without tranexamic acid administration.

The tranexamic acid relation appears to be less pronounced in older trauma patients than in younger patients (40). Patients with anticoagulation as premedication and tranexamic acid administration appear to have a survival advantage in the first 24 h after trauma, which disappears in terms of total mortality.

## Conclusion

Pre-existing anticoagulation in elderly patients has an impact on mortality after polytrauma. After tranexamic acid administration, a reduction in mortality was demonstrated compared to the calculated RISC II. A reduction in all-cause mortality for all patients at the age of >50 years could not be verified. A reduction in the 24 h-mortality could be demonstrated for patients with anticoagulation as premedication and tranexamic acid administration.

In propensity score matching, no higher complication rates were demonstrated in the tranexamic acid group. Despite lower hemoglobin and more mass transfusions, the tranexamic acid group was associated with a similar mortality rate.

## Limitations

This is a retrospective analysis of data provided by the TraumaRegister DGU^®^. Data of patients at the age of ≥50 years were included. Most studies define older trauma patients as above the age of 60, but different age limitations can be found in the literature. For our evaluation, we chose the age limit of 50 years because the share in anticoagulation as premedication increases significantly at this age. The risk of complications as thromboembolic events also increases from the age of 50.

We focused on pre-existing anticoagulation and donation of tranexamic acid. The information on anticoagulation as premedication and regular use was provided by the patient, family members, and the family doctor. Information on patient compliance is not documented in the TraumaRegister DGU^®^.

Multiple trauma patients who died before hospitalization were not included. Patients who were transferred after admission could not be included due to missing data. Only the data of patients up to discharge from the primary treating hospital were evaluated.

One pitfall of large trauma registries is that a complete data set is not available for every patient, so only existing data can be evaluated. Data on the ASA score and on anticoagulation as premedication were evaluated. Precise information about pre-existing conditions and additional prior medication is not documented in the TraumaRegister DGU^®^.

The included patients were treated by different emergency physicians and emergency teams, whose level of training and experience in emergency care was not considered.

For this reason, the results and conclusions on older trauma patients with anticoagulation as premedication and tranexamic acid administration are limited.

## Data availability statement

The original contributions presented in the study are included in the article/supplementary material, further inquiries can be directed to the corresponding author.

## Ethics statement

The presented study was approved by the local ethics committee of the medical faculty of Kiel University (D491/21). The publication is in line with the publication guidelines of the TraumaRegister DGU^®^ and registered as TR-DGU project ID-2020-043.

## Author contributions

SF-O: Conceptualization, Data curation, Investigation, Methodology, Writing – original draft, Writing – review & editing. GF: Conceptualization, Investigation, Writing – original draft. NK: Conceptualization, Investigation, Writing – original draft. RL: Data curation, Methodology, Writing – review & editing. SL: Methodology, Supervision, Writing – review & editing. OS: Methodology, Supervision, Writing – review & editing. TK: Methodology, Supervision, Writing – review & editing. MM: Methodology, Supervision, Writing – review & editing. AS: Supervision, Writing – review & editing. TraumaRegister DGU: Data curation, Writing – review & editing.

## TraumaRegister DGU

The TraumaRegister DGU® (TR-DGU) of the German Trauma Society (Deutsche Gesellschaft für Unfallchirurgie, DGU) is a multi-center database. The aim of the TraumaRegister DGU® is a pseudonymized and standardized documentation of severely injured patients.
